# Modeling the Effects of Nonpharmaceutical Interventions on COVID-19 Spread in Kenya

**DOI:** 10.1155/2020/6231461

**Published:** 2020-12-18

**Authors:** Duncan K. Gathungu, Viona N. Ojiambo, Mark E. M. Kimathi, Samuel M. Mwalili

**Affiliations:** ^1^Jomo Kenyatta University of Agriculture and Technology, Juja, Kenya; ^2^Machakos University, Machakos, Kenya; ^3^Strathmore University, Nairobi, Kenya

## Abstract

Mathematical modeling of nonpharmaceutical interventions (NPIs) of coronavirus disease (COVID-19) in Kenya is presented. A susceptible-exposed-infected-recovered (SEIR) compartment model is considered with additional compartments of hospitalized population whose condition is severe or critical and the fatality compartment. The basic reproduction number (*R*_0_) is computed by the next-generation matrix approach and later expressed as a time-dependent function so as to incorporate the NPIs into the model. The resulting system of ordinary differential equations (ODEs) is solved using fourth-order and fifth-order Runge–Kutta methods. Different intervention scenarios are considered, and the results show that implementation of closure of education institutions, curfew, and partial lockdown yields predicted delayed peaks of the overall infections, severe cases, and fatalities and subsequently containment of the pandemic in the country.

## 1. Introduction

On 7 January 2020, the World Health Organization (WHO) reported novel severe acute respiratory syndrome coronavirus (SARS-CoV-2) causative of the COVID-19 [1]. The WHO report details the chronology of how Wuhan in Hubei Province in China became a global epicenter of COVID-19 with the epidemiological link to the Huanan Seafood wet markets where there was sale of live animals. Over the period, COVID-19 has infected close to 26 million people worldwide with close to 1 million fatalities reported, necessitating the WHO to declare it a pandemic. With no viable vaccine at the moment, COVID-19 has initiated efforts focussed on containment and strategies aimed at reducing infections and fatalities. On the global platform, there has been a flurry of activity in determining the best strategy in the containment of the COVID-19 pandemic. A host of countries have instituted interventions not limited to social distancing, curfews, and total lockdowns. In [2], a model taking into consideration bats-hosts-reservoir-people transmission network was implemented, where reproduction numbers *R*_0_ were computed to determine the transmissibility of the virus between people and reservoirs. The value of *R*_0_ of reservoir to people was found to be 2.30 while that of people to people was 3.5. In [3], they delved into mathematical models of the COVID-19, and with a reproduction number of 2, they considered an SEIR model to model the effects of local social gatherings. With a 14-day infectious period, the model showed that, with 18-hour exposure, the attendees of the event had a protection efficacy of 70%. In [4], a conceptual SEIR model to gauge the effects of individual reactions and government reactions was investigated. This was in line with the Chinese government implementation of social distancing measures in areas considered as epicenters. The model incorporated zoonotic introductions and emigrations and showed that there were close relations with reported cases, even though asymptomatic transmission could not be established. In Ontario, Canada, Tuite et al. [5] implemented a mathematical model for transmission and mitigation strategies. They considered measures taken in the population which included quarantine, isolation of infectious cases, and hospitalization with intensive care unit (ICU) cases. It was reported that, in the absence of substantial physical social distancing and enhanced case detection and isolation, the ICU resources would be quickly overwhelmed. In [6], the authors considered a model for the prediction of new cases and made a comparison study of the United States of America, India, and Italy. Different scenarios under physical distancing measures such as lockdown were considered, and predictions were made using the available data. It was reported that, without mitigation of physical interaction of the populations, the peaks of infections would be realised much earlier in all the three countries.

Of late, the modeling efforts have advanced to cater for age-structured modeling to determine the transmission across different age strata. In [7], they use an age-structured SIR model with social contact matrices from surveys and Bayesian imputation to gauge the spread of the COVID-19 epidemic in India, where they use a generalization of the time-dependent *R*_0_ case study data, age distribution, and social contact structure. Their predictions implore on the duration of social distancing mitigation on such lockdowns and give a scope of when periodic ease of the lockdowns can be implemented. Further age-structured morbidity and mortality are reduced on implementation of these measures. Ivorra et al. [8] investigated a more complex variant of the SEIR referred to as *θ*-SEIHRD model to take into account the undetected infections and based their study in China. Their study showed that mitigation measures of limiting physical contact decrease the basic reproduction number, and subsequently, there is a reduction in infections. Further brisk detection is needed in order to reduce the number of infections. In [9], results of social distancing strategies for curbing the COVID-19 epidemic are presented. They simulate the reduction of reproduction numbers as a result of social distancing measures. In [10], an important parameter referred to as the identification parameter is included in the simulations to predict the peaks of the epidemic in Japan. This parameter is the ratio between the positive cases to the number of tests performed. Further, they used available data and the least-square-based procedure with Poisson noise to estimate the infection rate as a function of the identification rate, and they considered an SEIR compartmental model. They report that interventions have a positive effect in delaying the peak of the epidemic, and they propose longer interventions to contain the epidemic.

The results of the research studies outlined provide techniques knowledge on implementing a mathematical model for the epidemic in Kenya. Recently, in [11], the authors outlined the forecast of the scale of the COVID-19 epidemic in Kenya. In the study, they investigated the dissimilarities between China and Kenya in terms of demographics and geography. They forecasted the potential incidence rate and magnitude of the epidemic using the observed growth rate and age distribution of confirmed cases in China. They reported that the number of infected cases is likely to be high and that isolation of the asymptomatic infectious population will not be an adequate measure. Further, they proposed exceptional social distancing to “flatten the curve” and cautioned on the expected rebound of infections when the restrictions are lifted. In [12], an SEIR model was implemented taking into consideration the population's interaction with the environment. This was achieved by considering the environment as the host of SARS-CoV-2 virus. In the absence of social distancing, transmission and spread of COVID-19 was rife. As at 7 May 2020, Kenya had performed 28,002 tests, with 607 confirmed positive cases of COVID-19, 197 recoveries, and 29 fatalities [13]. The country's peak is not yet predicted and, in this paper, we investigate a variant of the SEIR model to predict the peaks of the infections, severity of the illness, and fatalities with and without the nonpharmaceutical measures.

## 2. Model Formulation

To model the mitigation efforts to curb the transmission and spread of COVID-19, we use the compartmental susceptible-exposed-infected-recovered model. We consider human-human transmission and divide the human population *N* at a time *t* into eight compartments. The susceptible population is denoted by *S*(*t*), the exposed population by *E*(*t*), the asymptomatic infectious population by *A*(*t*), the mild symptomatic population by *M*(*t*), the population who are severe and hospitalized by *H*(*t*), the critically ill population in the intensive care unit (ICU) by *C*(*t*), the recovered population by *R*(*t*), and the fatalities by *D*(*t*). Hence, the total human population is given as follows:

(1) $$\begin{array}{c} N\left(t\right)=S\left(t\right)+E\left(t\right)+A\left(t\right)+M\left(t\right)+H\left(t\right)+C\left(t\right)+D\left(t\right)+R\left(t\right). \end{array}$$

*S*(*t*) is the human population in Kenya that may be infected with (SARS-CoV-2) virus. The susceptible individuals move to *E*(*t*) after the incubation period to the onset of the disease. The exposed individuals move to either asymptomatic *A*(*t*) or the mild symptomatic *M*(*t*). Assuming the population in the asymptomatic have “immunocompetency,” they move to *R*(*t*). The population from *M*(*t*) can move either to *R*(*t*) or if their conditions deteriorate warranting hospitalization, they move to *H*(*t*) and are considered severe. The severe population on assessment can move to critical compartment *C*(*t*), and if their condition improves and becomes less critical, they are referred back to the general ward as severe cases. Further, on improved conditions, they recover and move to *R*(*t*). The population in *C*(*t*) who succumb move to *D*(*t*).

On 13 March 2020, after the first confirmed case, the Government of Kenya (GOK) instituted measures towards addressing the spread and transmission of COVID-19. The measures and interventions initially include a dusk-dawn curfew and massive campaign and sensitization measures of social and physical distancing. On 6 April 2020, the GOK extended and upscaled these measures with partial lockdowns in towns considered as hotspots in Kenya. These measures constituted enabled the formulation of assumptions for the development of the mathematical model. We assume the following:

- The disease is transmitted through human-human transmissions. There are no cross-infections occurring from pathogens in the environment nor human-animal transmissions.
- Susceptible (*S*) individuals are exposed/infected through contact with infectious individuals. Each infectious individual causes an average *R*_0_ secondary infection.
- After an average incubation period of 5.1 days, exposed (*E*) individuals either become asymptomatic (*A*) or exhibit mild infections (*M*), but not all infected persons exhibit symptoms.
- The virus-infected person is not infectious during the incubation period.
- Individuals with mild infection either recover (*R*) or worsen to a severe case (*H*).
- Individuals with severe infection either recover (*R*) or worsen to a critical case (*C*).
- Critically ill individuals either return to regular hospital or die.
- Demographics such as birth and death rates and immigration were not considered.
- Only a fraction of infective individuals can be identified by diagnosis.
- An infected individual acquires immunity upon recovery.

The flowchart below illustrates the compartmental SEIR model under consideration.

**Figure 1:**
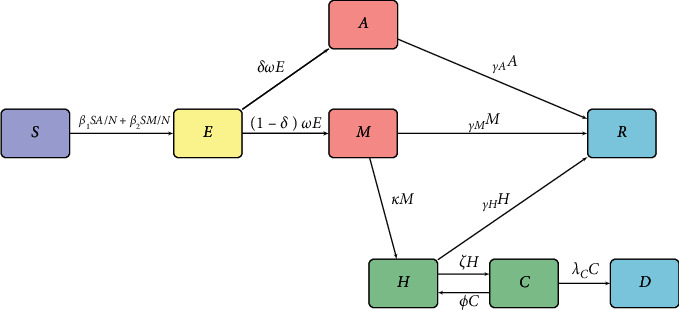
SEIR model flowchart.

The variant of the SEIR compartmental model [14, 15] illustrated in Figure 1 culminates to an eight-dimensional dynamical system of ordinary differential equations (ODEs) given by

(2) $$\begin{array}{c} \frac{\text{d}S}{\text{d}t}=-\left(\frac{\beta_{1}\text{SA}}{N}+\frac{\beta_{2}\text{SM}}{N}\right),\\ \frac{\text{d}E}{\text{d}t}=\left(\frac{\beta_{1}\text{SA}}{N}+\frac{\beta_{2}\text{SM}}{N}\right)-\omega E,\\ \frac{\text{d}A}{\text{d}t}=\delta \omega E-\gamma_{A}A,\\ \frac{\text{d}M}{\text{d}t}=\left(1-\delta \right)\omega E-\kappa M-\gamma_{M}M,\\ \frac{\text{d}H}{\text{d}t}=\kappa M+\phi C-\gamma_{H}H-\zeta C,\\ \frac{\text{d}C}{\text{d}t}=\zeta C-\phi C-\lambda_{C}C,\\ \frac{\text{d}R}{\text{d}t}=\gamma_{A}A+\gamma_{M}M+\gamma_{H}H,\\ \frac{\text{d}D}{\text{d}t}=\lambda_{C}C, \end{array}$$

with the initial conditions:

(3) $$\begin{array}{c} S\left(0\right)>0,E\left(0\right)>0,A\left(0\right)>0,M\left(0\right)>0,H\left(0\right)>0,C\left(0\right)>0,R\left(0\right)=0,D\left(0\right)>0. \end{array}$$

The parameters used in the model are given in Table 1.

The terms (*β*_1_SA/*N*) and (*β*_2_SM/*N*) describe the rate at which the susceptible population are infected by the asymptomatic population and the mild symptomatic, respectively.

In order to gauge the extent of the spread of COVID-19, experts have recommended extensive testing in the populations, but due to economic constraints in the country and globally, this testing has been below par. Therefore, many countries have resorted to introducing a wide range of NPIs to at least slow down the epidemic spread as they gather resources for mass testing and isolation of confirmed cases. In order to simulate and predict infection cases from the model, we use the framework developed by [10]. In this case, it is assumed only a fraction of the infected individuals are identified by the diagnosis. This fraction is given by *p* and it is referred to as the identification parameter which is 0 < *p* < 1. For this study, *p* is calculated as the ratio of the confirmed cases to the tests performed. Its usage in this study is as follows: suppose *S*+*E*+*A*+*M*+*H*+*C*+*R*+*D*=1, and one person is infected in Kenya of population 4.76 × 10^7^ at time *t*=0. Then, the infectious individuals *A*(0)+*M*(0) are given by Inf (0)=*p*(*A*(0)+*M*(0)) × 4.76 × 10^7^=1, and hence, *A*(0)+*M*(0)=1/(*p* × 4.76 × 10^7^). At any time *t*, the number of infectious individuals is given by Inf (*t*)=*p*(*A*(*t*)+*M*(*t*)) × 4.76 × 10^7^=1. Further, at *t*=0, if we assume *E*(0)=*H*(0)=*C*(0)=*R*(0)=*D*(0)=0, then *S*(0)=1 − *E*(0) − *A*(0) − *M*(0) − *H*(0) − *C*(0) − *R*(0) − *D*(0)=1 − 1/(*p* × 4.76 × 10^7^).

### 2.1. Existence of a Disease-Free Equilibrium (DFE)

To determine the DFE of the COVID-19 in Kenya, we solve the system of equations (2) after equating the right-hand side of the system to zeros. In this case, *A*=0, *M*=0,  and *H*=0, and subsequently, *C*=0, *R*=0, and *D*=0, which yields a DFE point as

(4) $$\begin{array}{c} \left(N,0,0,0,0,0,0,0\right). \end{array}$$

We compute the basic reproduction number *R*_0_ at DFE using the next-generation approach. Let *x*=(*E*, *A*, *M*)^*T*^ and d*x*/d*t*=*F*(*x*) − *V*(*x*), where

(5) $$\begin{array}{c} F\left(x\right)=\left(\begin{array}{c} \frac{\beta_{1}\text{SA}}{N}+\frac{\beta_{2}\text{SM}}{N}\\ 0\\ 0 \end{array}\right),\\ V\left(x\right)=\left(\begin{array}{c} \omega E\\ \gamma_{A}A-\delta \omega E\\ \left(\kappa +\gamma_{M}\right)M-\left(1-\delta \right)\omega E \end{array}\right). \end{array}$$

Obtaining the derivatives of *F*(*x*) and *V*(*x*) at DFE point yields **F** and **V** matrices as follows:

(6) $$\begin{array}{c} F=\left(\begin{array}{ccc} 0 & \beta_{1} & \beta_{2}\\ 0 & 0 & 0\\ 0 & 0 & 0 \end{array}\right),\\ V=\left(\begin{array}{ccc} \omega & 0 & 0\\ -\delta \omega & \gamma_{A} & 0\\ -\left(1-\delta \right)\omega & 0 & \kappa +\gamma_{M} \end{array}\right). \end{array}$$

The *R*_0_ is the spectral radius of the product of **FV**^−1^:

(7) $$\begin{array}{c} FV^{-1}=\left(\begin{array}{ccc} \frac{\beta_{1}\delta }{\gamma_{A}}+\frac{\beta_{2}\left(1-\delta \right)}{\kappa +\gamma_{M}} & \frac{\beta_{1}}{\gamma_{A}} & \frac{\beta_{2}}{\kappa +\gamma_{M}}\\ 0 & 0 & 0\\ 0 & 0 & 0 \end{array}\right), \end{array}$$

where *R*_0_ is given by

(8) $$\begin{array}{c} R_{0}=\frac{\beta_{1}\delta }{\gamma_{A}}+\frac{\beta_{2}\left(1-\delta \right)}{\kappa +\gamma_{M}}. \end{array}$$

This *R*_0_ consists of two terms showing transmission by the asymptomatic population and the mild symptomatic population.

### 2.2. Existence of Endemic Equilibrium (EE) Point

The endemic equilibrium point of model (2) is determined as positive steady state in which the COVID-19 is said to persist in a given population.

**Theorem 1 .**

The COVID-19 model has a unique endemic equilibrium point when *R*_0_ > 1; otherwise, the endemic equilibrium does not exist.

**Proof**

Suppose that (*S*^*∗*^, *E*^*∗*^, *A*^*∗*^, *M*^*∗*^, *H*^*∗*^, *C*^*∗*^, *R*^*∗*^, *D*^*∗*^) is a nontrivial equilibrium point of (2).

(9) $$\begin{array}{c} \left(\frac{\beta_{1}S^{*}A^{*}}{N}+\frac{\beta_{2}S^{*}M^{*}}{N}\right)=0, \end{array}$$

(10) $$\begin{array}{c} \left(\frac{\beta_{1}S^{*}A^{*}}{N}+\frac{\beta_{2}S^{*}M^{*}}{N}\right)-\omega E^{*}=0, \end{array}$$

(11) $$\begin{array}{c} \delta \omega E^{*}-\gamma_{A}A^{*}=0, \end{array}$$

(12) $$\begin{array}{c} \left(1-\delta \right)\omega E^{*}-\kappa M^{*}-\gamma_{M}M^{*}=0, \end{array}$$

(13) $$\begin{array}{c} \kappa M^{*}+\phi C^{*}-\gamma_{H}H^{*}-\zeta C^{*}=0, \end{array}$$

(14) $$\begin{array}{c} \zeta C^{*}-\phi C^{*}-\lambda_{C}C^{*}=0, \end{array}$$

(15) $$\begin{array}{c} \gamma_{A}A^{*}+\gamma_{M}M^{*}+\gamma_{H}H^{*}=0. \end{array}$$

From (11) and (12), we get

(16) $$\begin{array}{c} \omega E^{*}=\gamma_{A}A^{*}+\left(\kappa +\gamma_{M}\right)M^{*}. \end{array}$$

From (14), we get

(17) $$\begin{array}{c} C*\left(\zeta -\phi -\lambda_{C}\right)=0\Rightarrow \zeta =\phi +\lambda_{C}. \end{array}$$

From (15), we get

(18) $$\begin{array}{c} H^{*}=-\frac{1}{\gamma_{H}}\left(\gamma_{A}A^{*}+\gamma_{M}M^{*}\right). \end{array}$$

From (13), we get

(19) $$\begin{array}{c} \left(\phi -\zeta \right)C^{*}=\gamma_{H}H^{*}-\kappa M^{*}. \end{array}$$

Using (18) in (19) yields

(20) $$\begin{array}{c} C^{*}=-\frac{\left(\gamma_{A}A^{*}+\gamma_{M}M^{*}+\kappa M^{*}\right)}{\phi -\zeta }. \end{array}$$

From (15), we get

(21) $$\begin{array}{c} S^{*}\left(\frac{\beta_{1}A^{*}}{N}+\frac{\beta_{2}M^{*}}{N}\right)=\gamma_{A}A^{*}+\left(\gamma_{M}+\kappa \right)M^{*}\\ \Rightarrow S^{*}=\frac{N\left(\gamma_{A}A^{*}+\left(\gamma_{M}+\kappa \right)M^{*}\right)}{\left(\beta_{1}A^{*}+\beta_{2}M^{*}\right)}. \end{array}$$

Both *A*^*∗*^ and *M*^*∗*^ should satisfy

(22) $$\begin{array}{c} \frac{\beta \text{SI}}{N}=\omega E, \end{array}$$

(23) $$\begin{array}{c} E=\gamma I. \end{array}$$

Using (23) in (22) yields

(24) $$\begin{array}{c} S=\frac{N\omega \gamma }{\beta }. \end{array}$$

Using (23) in (16) yields

(25) $$\begin{array}{c} \omega \gamma I=\gamma_{A}A^{*}+\left(\kappa +\gamma_{M}\right)M^{*}. \end{array}$$

Using (24) in (21) yields

(26) $$\begin{array}{c} \frac{N\omega \gamma }{\beta }=\frac{N\left(\gamma_{A}A^{*}+\left(\gamma_{M}+\kappa \right)M^{*}\right)}{\left(\beta_{1}A^{*}+\beta_{2}M^{*}\right)},\\ \left(\omega \gamma \beta_{1}-\beta \gamma_{A}\right)A^{*}+\left(\omega \gamma \beta_{2}-\beta \left(\gamma_{M}+\kappa \right)\right)M^{*}=0\\ \Rightarrow \gamma_{A}A^{*}+\left(\kappa +\gamma_{M}\right)M^{*}=\omega \gamma I. \end{array}$$

An endemic scenario *I* ≈ *αN*, where *α* is the attack rate, since almost everyone gets infected.

Therefore, solving the following equations simultaneously yields *A*^*∗*^ and *M*^*∗*^:

(27) $$\begin{array}{c} \left(\omega \gamma \beta_{1}-\beta \gamma_{A}\right)A^{*}+\left(\omega \gamma \beta_{2}-\beta \left(\gamma_{M}+\kappa \right)\right)M^{*}=0,\\ \gamma_{A}A^{*}+\left(\kappa +\gamma_{M}\right)M^{*}=\omega \gamma \alpha N. \end{array}$$

Hence,

(28) $$\begin{array}{c} A^{*}=\frac{\omega \gamma \beta_{2}-\beta \left(\gamma_{M}+\kappa \right)\omega \gamma \alpha N}{\left(\gamma_{A}\left(\omega \gamma \beta_{2}-\beta \left(\gamma_{M}+\kappa \right)\right)\right)-\left(\left(\kappa +\gamma_{M}\right)\left(\omega \gamma \beta_{1}-\beta \gamma_{A}\right)\right)},\\ M^{*}=\frac{\left(\omega \gamma \beta_{1}-\beta \gamma_{A}\right)\omega \gamma \alpha N}{\left(\omega \gamma \beta_{1}-\beta \gamma_{A}\right)\left(\kappa +\gamma_{M}\right)-\gamma_{A}\left(\omega \gamma \beta_{2}-\beta \left(\gamma_{M}+\kappa \right)\right)}. \end{array}$$

### 2.3. Prediction of Infection Cases

In the quest to determine the level of preparedness to curb the spread of COVID-19, it is important to predict future numbers of daily infection cases and cumulative infections. This provides a basis for policymakers to know when the available healthcare infrastructure is likely to be overwhelmed. In this section, we used a Metropolis–Hastings Markov chain Monte Carlo (MCMC) algorithm for nonlinear Gaussian functions to estimate the daily and cumulative cases from Kenya's data of daily confirmed cases. Using 20,000 iterations of the MCMC procedure, we are able to predict the daily infection cases and the cumulative infections for a period of 90 days.

**Figure 2:**
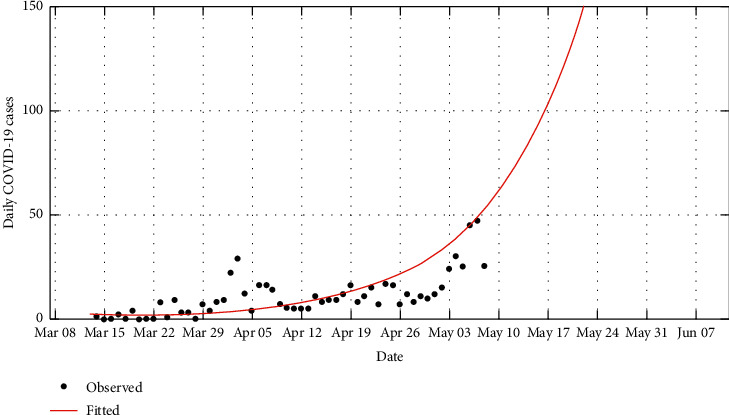
Prediction of daily cases over a 90-day period.

**Figure 3:**
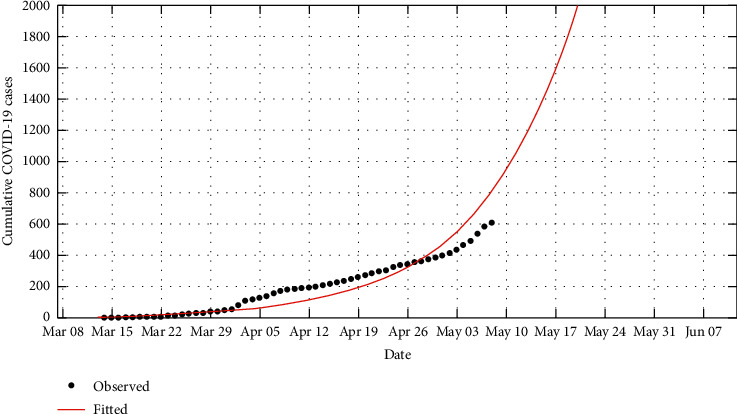
Prediction of the cumulative cases over a 90-day period.

Using the available daily data of the COVID-19 cases [13], we are able to use these procedures to predict the daily infection cases as shown in Figure 2 and when the country is likely to have reported the first 1,000 infection cases from Figure 3.

## 3. Simulation and Results

**Figure 4:**
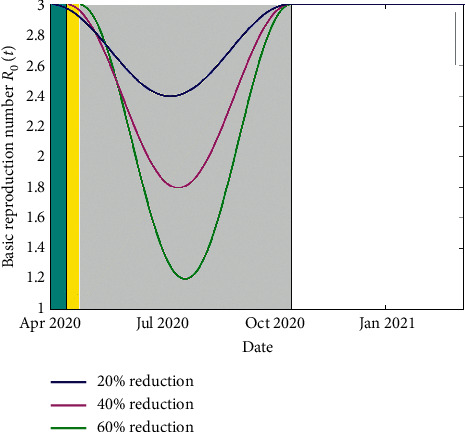
Depiction of the effects of school closedown, curfew, and partial lockdown on the time-dependent basic reproduction number. The duration of school closedown, of 210 days, is indicated by the cyan color. However, it is overlapped by the duration of curfew implementation, shown here in yellow. The yellow region is in turn overlapped by the gray region which represents the duration of partial lockdown implementation.

In this section, we report on the results of simulations of the COVID-19 model. The model's equation (2) is solved using a fourth- and fifth-order Runge–Kutta method, which is implemented in MATLAB. We consider *N*=4.76 × 10^7^ people which is Kenya's total population as per the 2019 census. The simulations are done using the parameters listed in Table 1. The human-human contact reduction measures we consider are school closedown, dusk-to-dawn curfew, and partial lockdowns in towns and cities perceived as COVID-19 hotspots. The essence of contact reduction is to mitigate the interaction between the susceptible and infectious populations. Based on studies of COVID-19 spread in China, Italy, and Spain, we suppose that if no mitigation measures are in place, then an infectious individual would infect three secondary cases, in his/her interaction sphere. Thus, we consider the *R*_0_ as 3, for the unmitigated scenario. The first case of the novel coronavirus was confirmed in Kenya on 13 March 2020. On 16 March 2020, schools were closed, which was the first NPI that was implemented by the Kenyan government. Then 14 days into the school closedown, curfew measures were implemented. Thereafter 24 days into school closedown, partial lockdown was implemented. In our simulations, we implement the school closure for 210 days, curfew for 196 days, and partial lockdown for 186 days. This is done via a time-dependent *R*_0_, which was taken as a cosine function (see [9]). We presume that school closure yields a 20% reduction in contact while adding a curfew on to the school closure results in a 40% reduction, and introduction of the partial lockdown on to the other two NPIs yields a 60% reduction in contacts as shown in Figure 4.

**Figure 5:**
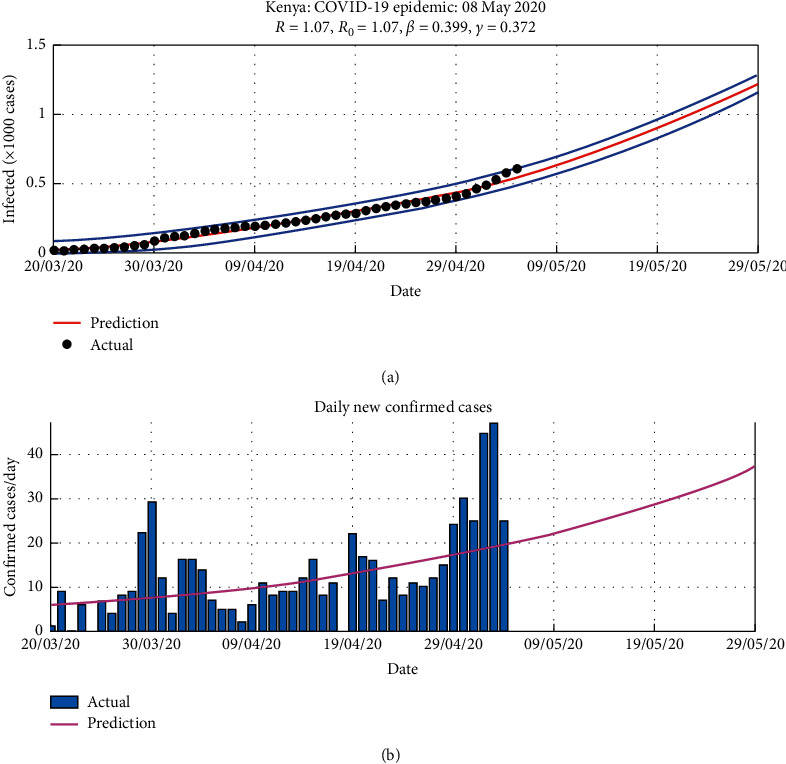
The basic reproduction number *R*_0_ is used to validate the results from the model. It shows that, in the period of simulations, the models' *R*_0_ in Figure 4 collates with the *R*_0_ of between 1.1 and 2 reported for the COVID-19 epidemic in Kenya.

A reduction in *R*_0_ implies that fewer individuals are being infected, and hence, the disease spread is slowed down. In Figure 5, predictions of the daily confirmed cases and the prediction of the cumulative infections are given which shows that, with a basic reproduction number of greater than 1, the infections shall continue increasing.

**Figure 6:**
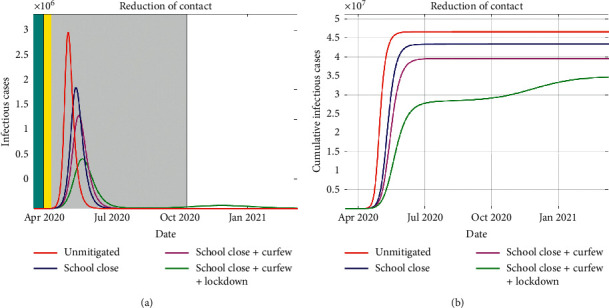
The simulated daily cases of infected individuals is shown in (a), whereby the duration of NPIs implementation is also indicated by the colored regions. The cumulative cases of the infected individuals are depicted in (b). The NPIs are shown to effectively mitigate the spread of the epidemic in the population. A combination of the three measures is the most effective strategy in that even though a new wave of infection emerges, it flattens out with a much lower peak.

In Figures 6(a) and 6(b), we report on the simulation of infections and the cumulative infections under different scenarios. These different scenarios are when there is no mitigation, when the schools are closed, when schools are closed and dusk-dawn curfew is instituted, and when schools are closed, curfew is in place, and partial lockdown is in place.

From the figures, we deduce that the NPIs instituted aid in containment of the epidemic spread, resulting in fewer people being infected as compared with the unmitigated scenario. In particular, we see a much higher peak of infections in the unmitigated scenario, which arrives much faster due to the high rate of infections when *R*_0_ = 3.0. For the first two NPIs, the infection peaks are lower and come at a later date. When all the three NPIs are combined, resulting in *R*_0_ of 1.2, the infections peak at a much lower value and the epidemic begins to subside. However, this leaves a large population in the susceptible compartment such that when the value of *R*_0_ begins to rise, towards the end of the NPI duration, a rebound of infections occurs. Due to the effectiveness of the combined NPIs, the new peak is much lower and the epidemic is eradicated. The measures result in a reduction in the cumulative number of infections (see Figure 6(b)). Combining all the three NPIs leads to a significant delay of the epidemic spread, a time the health system can utilize to put mechanisms and facilities to respond to the epidemic appropriately.

**Figure 7:**
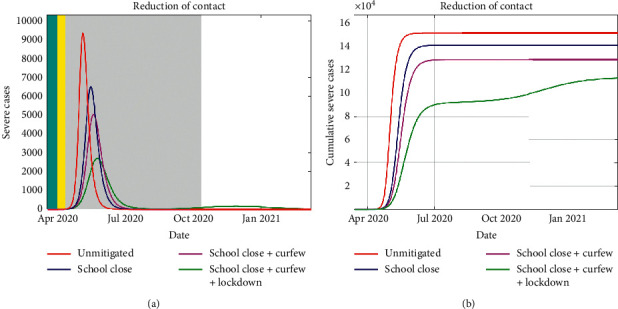
Over the duration of the implementation of the NPIs, the daily severe cases are shown in (a) where the duration of these interventions is indicated by the colored region. In (b), the cumulative severe cases for the three mitigation measures are shown. It shows that the implementation of the three measures leads to a reduced number of severe cases.

In Figures 7(a) and 7(b), we report on the number of severe cases of COVID-19 who are hospitalized. Reduction of the severe cases is important as this represents the proportion of the population who are directly utilizing the healthcare facilities at a given time. Due to limited hospital infrastructure and resources, delay of this peak is vital for the health systems to prepare for a guaranteed influx of patients in need of medical attention. Furthermore, any reduction of the hospitalized results in significant reduction of severe cases transfer to critical cases that require ICU services.

From Figures 7(a) and 7(b), we deduce that stringent implementation of the NPIs is key in ensuring the available hospital infrastructure is not overwhelmed during the epidemic. The national government and county governments under the Health Ministry directive have boosted the bed capacity at all hospital levels, including establishing isolation centers. If schools are closed, curfew is implemented and lockdown is instituted, the simulation shows that the hospitalized population may not overwhelm the established healthcare infrastructure capacity.

**Figure 8:**
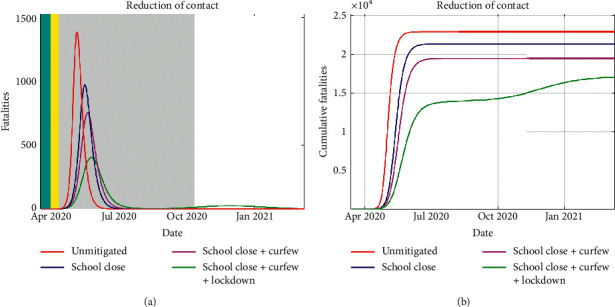
The simulated daily number of fatalities is shown in (a), where the duration of the NPIs is indicated by the colored region. The cumulative number of fatalities is presented in (b). In the event all three mitigation measures are in place, the cumulative cases “plateau” at a much lower peak.

In Figures 8(a) and 8(b), we present the results of simulations of fatalities under different intervention scenarios.

Obviously, fatalities from COVID-19 will have the highest peak if NPIs are not instituted. From Figure 8(a), delayed peaks are realised if the NPIs are in place and the best scenario is if the considered measures are combined, for the considered duration of implementation. A rebound of infections also results in a resurgence of fatalities due to the severity of the disease, especially among the older population.

## 4. Conclusion

Since COVID-19 vaccine development will take a significantly longer period of time before it becomes globally available, it is imperative on all medical sectors in the country to adhere to mitigation measures and intervention policies set by the government. The implementation of school closure, dusk-to-dawn curfew, and partial lockdown is the measure in curbing the spread of COVID-19 that are effective and lead to the flattening of the curve within the duration of implementation. Relaxation of the mitigation measures on or before September 2020 will likely lead to a resurgence and the country may experience a new wave of infections. Devoid of the aftershocks of these interventions, the country will have no infections as at 31 December 2020. This model developed at the advent of the COVID-19 pandemic demonstrates the projections of infections and fatalities as a result of the pandemic in Kenya. It forms a basis of determination of hospital needs estimates of the country which raises the level of preparedness in case of a second wave of infections.

## Figures and Tables

**Table 1:** Parameters of the SEIR model of COVID-19.

Model parameter name	Symbol	Value	References
Rate of infection from the asymptomatic cases (*A*)	*β*_1_	4.0	Estimated
Rate of infection from the mild cases (*M*)	*β*_2_	2.667	Estimated
Proportion of asymptomatic cases	*δ*	85%	[2, 12, 16]
Proportion of critical cases (*C*) that progress back to severe cases (*H*)	*ϕ*	0.5	[2, 12]
Proportion of severe cases (*H*) that progress to critical cases (*C*)	*ζ*	0.302	[12]
Reciprocal of the average incubation period	*ω*	0.196	[12]
Recovery proportion of asymptomatic cases (*A*)	*γ*_*A*_	1.0	[12, 16]
Recovery proportion of severe cases (*H*)	*γ*_*H*_	0.698	[9, 12, 17]
Recovery proportion of mild cases (*M*)	*γ*_*M*_	0.9815	[12, 16]
Proportion of mild cases (*M*) that progress to severe cases (*H*)	*κ*	0.0185	[12, 16]
Proportion of fatalities of critical cases (*C*)	*λ*_*C*_	0.5	[12, 18]

## Data Availability

The data used to support the findings of this study are available from the corresponding author upon request.
